# γδ T Cells and NK Cells – Distinct Pathogenic Roles as Innate-Like Immune Cells in CNS Autoimmunity

**DOI:** 10.3389/fimmu.2015.00455

**Published:** 2015-09-08

**Authors:** Sarah C. Edwards, Aoife M. McGinley, Niamh C. McGuinness, Kingston H. G. Mills

**Affiliations:** ^1^Immune Regulation Research Group, Trinity Biomedical Sciences Institute, School of Biochemistry and Immunology, Trinity College Dublin, Dublin, Ireland; ^2^Trinity College Institute of Neuroscience, Trinity College Dublin, Dublin, Ireland

**Keywords:** γ δ T cells, NK cells, IL-17, multiple sclerosis, experimental autoimmune encephalomyelitis, autoimmunity

## Introduction

Multiple sclerosis (MS) is a chronic inflammatory, demyelinating disease that affects the central nervous system (CNS) resulting in progressive cognitive decline and physical disability. Experimental autoimmune encephalomyelitis (EAE) is an animal model of MS that has been used to understand the cellular and molecular mechanisms underlying CNS inflammation and autoimmunity. Since the discovery of IL-17-sereting CD4^+^ T cells (Th17 cells) over 10 years ago, these cells have been the main focus of attention as mediators of pathology in MS and EAE (1, 2). However, in recent years evidence has emerged that lymphocytes with innate-like properties are potent producers of IL-17 and related pro-inflammatory cytokines (3–6). γδ T cells, NKT, and innate lymphoid cells have been shown to be major sources of IL-17 in host control of a variety of bacterial, viral, and fungal infections. However, dysregulation of these innate-like lymphocytes can also result in severe pathology in EAE and other models of autoimmunity. The role of IFN-γ in the pathogenesis of autoimmune diseases is more controversial. Like Th17 cells, transfer of myelin antigen-specific Th1 cells can induce EAE in naïve mice (7, 8). However, IFN-γ, the signature cytokine of Th1 and natural killer (NK) cells, has been shown to inhibit the function of pathogenic Th17 cells, as well as promoting development of encephalitogenic T cells during induction of EAE (8, 9). Immunotherapeutics that suppress the induction or function of Th17 cells have proved successful in treating psoriasis, but have had more variable success in MS patients (10). Based on recent studies on the role of innate-like lymphocytes in the pathogenesis of EAE, we propose that these cells may provide more selective and improved drug targets for the treatment of MS.

## CD4^+^ T Cells

Th1 cells were originally thought to be the main pathogenic cells in MS and EAE. This was in part attributed to the fact that IL-12p40^−/−^ mice were resistant to EAE, and treatment of MS patients with IFN-γ exacerbated disease (11). However, mice deficient in IFN-γ or T-bet, which lack Th1 cells, were not protected from EAE (12, 13). The discovery of IL-23 partly resolved this paradox. IL-23 and IL-12 share a common p40 chain, which associates with a separate p19 chain to make IL-23 or with a p35 chain to make IL-12. Like IL-23p40^−/−^ mice, IL-23p19^−/−^ mice are resistant to EAE, whereas IL-12p35^−/−^ mice are susceptible (14). IL-23 was then shown to be essential in driving the induction or the expansion of IL-17-secreting CD4^+^ T cells, which were termed Th17 cells (15–17). IL-17-producing Th17 cells proved to have a key role in inflammation and autoimmunity when they were found capable of transferring EAE to naive mice (16).

In addition to IL-17A, Th17 cells produce an array of other inflammatory cytokines, including IL-17F, GM-CSF, IL-22, IL-21, IL-26 and TNF-α (16, 18–24). Since their discovery, Th17 cells have been implicated in the pathogenesis of most common autoimmune diseases, including psoriasis, rheumatoid arthritis (RA), and MS, and in animal models of these diseases. Despite the extensive studies on Th17 cells, the relative roles of Th1 and Th17 cells in the pathogenesis of MS and other autoimmune diseases are still unclear. Data from our laboratory and others show that both Th1- and Th17-polarized T cells are capable of transferring EAE (7, 8). Furthermore, CD4^+^ T cells secreting both IL-17 and IFN-γ are detectable in the CNS of mice with EAE (25–27). Therefore, it is our opinion that both Th1 and Th17 cell subsets play important roles in autoimmune pathology, but that there is plasticity between these T cell types and that the pathogenic function of other immune cells, especially cells of the innate immune system should not be ignored.

## γ δ T Cells

**Figure 1:**
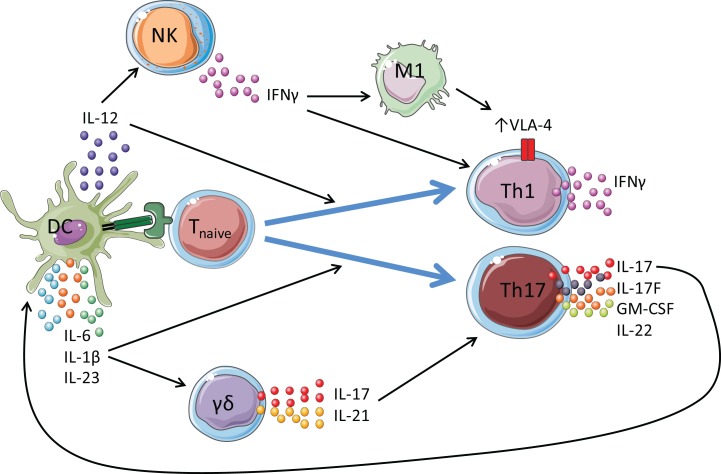
**Proposed roles for ***γ*** ***δ*** T cells and NK cells in amplifying pathogenic CD4^+^ T cell responses in EAE**. Dendritic cells (DCs) are activated by TLR and NLR agonists. Antigenic peptide is presented by MHC class II molecules on DCs to the TCR on T cells. This, along with co-stimulatory signals, activates the T cell. Once activated, DCs secrete cytokines including IL-1β, IL-6, IL-23, and IL-12 that promote the polarization of naïve T cells into effector cells. IL-12 promotes the induction of Th1 cells, which are primarily IFN-γ producers. IL-1β, IL-6, and IL-23 promote the differentiation and expansion of Th17 cells, which secrete IL-17 (and IL-22, GM-CSF, IL-21) and mediate protection against extracellular pathogens, such as fungi, and are heavily implicated in the pathology of autoimmune diseases. γ δ T cells secrete IL-17 and IL-21 following stimulation with IL-1β and IL-23 without TCR engagement, which act in an autocrine loop to promote further IL-17 production by Th17 cells in the development of EAE. NK cells provide an early source of IFN-γ to drive VLA-4 expression on Th1 and Th17 cells, allowing these cells to traffic from the peripheral lymphoid organs and into the CNS.

γδ T cells represent around 2–5% of peripheral lymphocytes and are known to play an important role in innate and adaptive immunity at mucosal surfaces. γδ T cells have been described as polyfunctional; they produce an array of cytokines, including IL-17A, IL-17F, IFN-γ, IL-10, IL-22, IL-21, GM-CSF, and TNF-α (28–31). The IL-17-producing γδ T cells share many features with CD4^+^ Th17 cells, including expression of RORγt, IL-1R1, IL-23R, and CCR6 (32). Although γδ T cells do express a unique T cell receptor (TCR), engagement of this TCR with MHC-antigen complexes is not a prerequisite for their activation. Unlike conventional αβ T cells, cytokine stimulation alone is sufficient for activation of IL-17-secreting γδ T cells, making these cells rapid and potent mediators of inflammation (28). γδ T cells have been shown to be pathogenic in a variety of autoimmune diseases, such as EAE, collagen-induced arthritis (CIA), and most recently in EAU (33–35). Before the discovery of Th17 cells and their signature cytokine IL-17, it was assumed that early IFN-γ derived from γδ T cells was the main pathogenic cytokine driving EAE; this was in part based on the established role of IFN-γ-secreting γδ T cells in enhancing CD4^+^ and CD8^+^ T cell responses in anti-tumor immunity (36). However, our studies, supported by recent results from other labs, suggest that the pathogenic function of γδ T cells is mediated by their production of IL-17 and related cytokines, including IL-21 and GM-CSF (28). γδ T cells can secrete IL-17 in response to IL-1, IL-18, and IL-23 without TCR engagement, promoting the induction of Th1 and Th17 cells and amplifying their encephalitogenic function during the development of EAE (28, 37, 38) (Figure 1). Studies from our group have demonstrated that dendritic cells (DCs) can enhance the ability of IL-1- and IL-23-activated γδ T cells to promote IL-17 production by Th17 cells (28). Furthermore, DCs express IL-17R and secrete IL-23 in response to IL-17, which was enhanced by LPS and blocked by anti-IL-17R. These findings suggest that γδ T-cell-derived IL-17 may act in a positive feedback loop involving DC activation leading to enhanced Th17 cell effector function during EAE. *In vitro* studies have also suggested a pathogenic role for γδ T cells in demyelinating diseases of the CNS, as γδ T cells are indirectly responsible for axonal demyelination through toxic destruction of oligodendrocytes, cells responsible for myelinating axons (39).

Importantly, data from our laboratory and others have shown that γδ T cells infiltrate the brain and spinal cord in large numbers during the course of EAE, where they produce IL-17 and related cytokines (28, 37, 38). Vγ4^+^ T cells were identified as the main IL-17-producing γδ T cell in the brains of mice with EAE, but Vγ1 and Vγ6 T cells are also present (28). Vγ4^+^ T cells are also key players in a variety of other autoimmune conditions, such as myocarditis, (40) psoriasis (41), and CIA (34). The pathogenic role of γδ T cells in EAE was demonstrated by a reduction in disease severity in TCRδ^−/−^ mice (42). Furthermore, studies in the relapsing-remitting EAE model showed a significant reduction in clinical severity when mice were treated with a TCRδ depleting antibody immediately before disease onset or during the chronic phase of disease (33). In addition, experiments in the adoptive transfer model of EAE demonstrated that depletion of γδ T cells reduced clinical severity and delayed the onset of disease (43).

The pivotal role of γδ T cells in the pathogenicity of EAE is also reflected in MS, where clonal expansion of γδ T cells has been observed in the cerebrospinal fluid (CSF) of patients with recent disease onset (44). Furthermore, an increased frequency of γδ T cells have been detected in the peripheral blood of patients with MS (45) and an accumulation of γδ T cells has been described in acute brain lesions (46). Based on these findings, we propose that γδ T cells have a critical role in the active stages of both EAE and MS.

## NK Cells

Natural killer cells are innate lymphocytes named for their cytolytic activity, which can control tumor growth and microbial infection. NK cells can produce the pro-inflammatory cytokines IFN-γ and TNF-α, as well as the immunosuppressive cytokine IL-10 and the growth factor GM-CSF in response to IL-12, IL-15, or IL-18 (47).

Human NK cells can be broadly separated into two types on the basis of their expression of CD16 and CD56. CD16^+^CD56^dim^ cells express more intracellular perforin and are more efficient killers, whereas the CD16^dim/−^CD56^bright^ subset produce greater amounts and a wider variety of cytokines, and are more regulatory in nature (48). The general consensus in the literature is that the CD56^bright^ NK cells play a protective role in MS. It has been reported that the ratio of CD56^bright^:CD56^dim^ cells is higher in the CSF of MS patients relative to control subjects (49). Furthermore, the CD56^bright^ subtype is expanded in response to the MS disease modifying therapies IFN-β (50), daclizumab (51, 52) and natalizumab (53). There is also an established link between disease relapse and a decrease in the number of total circulating NK (CD16^+^CD56^+^) cells in peripheral blood of MS patients (54). Conversely, an increase in NK cell number and migratory capacity has been associated with remission (55). Therefore, it is possible that certain subsets of NK cells may have a role in controlling CNS inflammation in MS patients.

A potential mechanism underlying the protective effect of NK cells in MS was provided by the observation that these CD56^bright^ NK cells can kill activated, but not resting, autologous CD4^+^ T cells by inducing apoptosis through degranulation (56). While less is known about the role of the CD56^dim^ subset in MS, the frequency of these cells in the circulation is enhanced in the progressive forms of the disease, (57) a phenomenon which also occurs with age (58). Therefore, it is possible that CD56^dim^ NK cells may contribute to neurodegeneration, however, further investigation is required to confirm this hypothesis.

Studies in the animal model EAE have generated more extensive data on the role of NK cells that has led to more controversy. The severity of EAE is enhanced in mice deficient in fractalkine receptor expression, which is required for NK cell recruitment to the inflamed CNS (59), suggesting that NK cells may play a role in limiting CNS inflammation. This is consistent with a more recent publication suggesting that a population of CNS-resident NK cells have a protective role in EAE through suppression of myelin-reactive Th17 cells (60). By contrast, IFN-γ from NK cells has also been shown to promote autoreactive Th1 responses and contribute to the pathogenesis of EAE (61).

Depletion of NK cells in EAE using either anti-NK1.1 or anti-asialo GM1, which induce apoptosis (62) or complement-dependent lysis (63), respectively, has generated conflicting reports of both exacerbation (60, 64–66) and amelioration (61, 67) of clinical disease. These discrepancies may reflect differences in the antibodies used, the depletion regimen, and a focus on disease peak. Data from our laboratory suggest that NK cells have a pathogenic role in disease induction; NK cells were found to infiltrate the CNS of mice with EAE before the onset of clinical symptoms, and depletion of these cells at this early time-point led to a significant reduction in disease severity (8). The pathogenic role of NK cells was attributed to early IFN-γ production, as early depletion of NK cells did not affect the clinical course of EAE in IFN-γ^−/−^ mice. IFN-γ from NK cells polarized macrophages to an M1 phenotype and thus conferred encephalitogenic potential on CD4^+^ T cells by upregulating expression of the integrin VLA-4, which is required for CD4^+^ T cell infiltration into the CNS (8) (Figure 1). We believe that NK cells play a critical pathogenic role in EAE by acting as an early source of innate IFN-γ in the initiation of disease. However, late in disease, IFN-γ production by Th1 cells, activated by NK cells, may have protective role through suppression of cytokine production by Th17 cells. This might explain the finding in MS patients of an association between reduced NK cells numbers and disease relapse (54) and increased NK cells and disease remission (55).

## Conclusion

Understanding the Th17/IL-17 axis in both protective and dysregulated immunity has led to the development of many promising front line therapies for autoimmune diseases. However, we believe that research in this area has been too heavily focused on CD4^+^ T cells and that further study on innate immunity may provide vital insight into mechanisms of disease and improved therapies. Although much of the attention has been on Th17 cells, these are not the only source of the pro-inflammatory cytokine IL-17. Innate-like lymphocytes, such as γδ T cells and NK cells, provide an early source of IL-17 and IFN-γ, traffic to the CNS early during development of EAE, and provide an amplification loop for the activation of pathogenic CD4^+^ T cells (Figure 1). IL-17 and IL-21 derived from γδ T cells enhances the pathogenicity of Th17 cells in EAE. Furthermore, IFN-γ derived from NK cells polarizes M1-type macrophages and enhances the encephalitogenic activity of CD4^+^ T cells by upregulating VLA-4 expression. Treatment of MS patients with biological drugs designed to suppress the induction, migration, or function of CD4^+^ T cells, such as natalizumab, come with an increased risk of infection, in particular progressive multifocal leukoencephalopathy (PML) (68). Given the important role of small populations of γδ T cells and NK cells in the pathogenesis of EAE, we propose that a better understanding of the activation and function of these innate-like lymphocytes and their secreted cytokines may lead to new and more selective therapeutic interventions for the treatment of MS.

## Conflict of Interest Statement

The authors declare that the research was conducted in the absence of any commercial or financial relationships that could be construed as a potential conflict of interest.
